# Clinical Parameters Are More Likely to Be Associated with Thyroid Hormone Levels than with Thyrotropin Levels: A Systematic Review and Meta-Analysis

**DOI:** 10.1089/thy.2019.0535

**Published:** 2020-12-07

**Authors:** Stephen P. Fitzgerald, Nigel G. Bean, Henrik Falhammar, Jono Tuke

**Affiliations:** ^1^ Department of General Medicine and Royal Adelaide Hospital, Adelaide, South Australia.; ^2^ Department of Endocrinology, Royal Adelaide Hospital, Adelaide, South Australia.; ^3^ School of Medicine, University of Adelaide, Adelaide, South Australia.; ^4^ School of Mathematical Sciences, University of Adelaide, Adelaide, South Australia.; ^5^ ARC Centre of Excellence for Mathematical and Statistical Frontiers, University of Adelaide, Adelaide, South Australia.; ^6^ Department of Endocrinology, Metabolism and Diabetes, Karolinska University Hospital, Stockholm, Sweden.; ^7^ Department of Molecular Medicine and Surgery, Karolinska Institutet, Stockholm, Sweden.; ^8^ Wellbeing and Chronic Preventable Diseases Division, Menzies School of Health Research and Royal Darwin Hospital, Tiwi, Australia.

**Keywords:** thyroid hormones, TSH, clinical parameters, correlation, subclinical thyroid dysfunction

## Abstract

***Background:*** Though the functional states of other endocrine systems are not defined on the basis of levels of controlling hormones, the assessment of thyroid function is based on levels of the controlling hormone thyrotropin (TSH). We, therefore, addressed the question as to whether levels of thyroid hormones [free thyroxine (fT4), total triiodothyronine (TT3)/free triiodothyronine (fT3)], or TSH levels, within and beyond the reference ranges, provide the better guide to the range of clinical parameters associated with thyroid status.

***Methods:*** A PubMed/MEDLINE search of studies up to October 2019, examining associations of levels of thyroid hormones and TSH, taken simultaneously in the same individuals, with clinical parameters was performed. We analyzed atrial fibrillation, other cardiac parameters, osteoporosis and fracture, cancer, dementia, frailty, mortality, features of the metabolic syndrome, and pregnancy outcomes. Studies were assessed for quality by using a modified Newcastle–Ottawa score. Preferred Reporting Items for Systematic Reviews and Meta-analyses guidelines were followed. A meta-analysis of the associations was performed to determine the relative likelihood of fT4, TT3/fT3, and TSH levels that are associated with the clinical parameters.

***Results:*** We identified 58 suitable articles and a total of 1880 associations. In general, clinical parameters were associated with thyroid hormone levels significantly more often than with TSH levels—the converse was not true for any of the clinical parameters. In the 1880 considered associations, fT4 levels were significantly associated with clinical parameters in 50% of analyses. The respective frequencies for TT3/fT3 and TSH levels were 53% and 23% (*p* < 0.0001 for both fT4 and TT3/fT3 vs. TSH). The fT4 and TT3/fT3 levels were comparably associated with clinical parameters (*p* = 0.71). More sophisticated statistical analyses, however, indicated that the associations with TT3/fT3 were not as robust as the associations with fT4.

***Conclusions:*** Thyroid hormones levels, and in particular fT4 levels, seem to have stronger associations with clinical parameters than do TSH levels. Associations of clinical parameters with TSH levels can be explained by the strong negative population correlation between thyroid hormones and TSH. Clinical and research components of thyroidology currently based on the measurement of the thyroid state by reference to TSH levels warrant reconsideration.

## Introduction

Thyroid function testing (1,2) and monitoring (3) are based on the measurement of thyrotropin (TSH) levels. Patients are therefore classified as having euthyroidism (normal TSH and thyroid hormone levels), overt thyroid dysfunction (abnormal TSH and thyroid hormone levels), subclinical thyroid dysfunction (abnormal TSH/normal thyroid hormone levels), and isolated hyper/hypothyroxinemia (normal TSH/abnormal thyroid hormone levels).

This classification of thyroid function is based on the concept of TSH levels being the most sensitive indicator of thyroid function such that subclinical thyroid dysfunction as currently defined is believed to be more significant than isolated hyper/hypothyroxinemia (2), as indicated by the alternative term for the latter, “euthyroid hyper/hypothyroidism” (4).

Subclinical thyroid dysfunction is common and comprises most cases of thyroid dysfunction with a population prevalence of ∼5% (5–9), increasing to 15% in older adults (9). Even though it is generally asymptomatic or associated only with non-specific symptoms, subclinical thyroid dysfunction has been associated with many adverse outcomes across a variety of organ systems (5–9). Therefore, despite the lack of convincing evidence of significant benefit (10,11), treatment for subclinical thyroid dysfunction has been recommended in certain circumstances (6–9).

It has previously been suggested by some authors that the earlier definition of subclinical thyroid dysfunction is overly simple and that its diagnosis should not be based solely on the TSH level being outside of a general population range (12,13). Rather, it is claimed that more accuracy may be achieved by defining a normal reference range for the combination of thyroid hormones and TSH.

However, any model whereby judgment of the thyroid status includes consideration of the TSH level is anomalous, in that the levels of other physiological parameters are not judged by the levels of their controlling hormones. For example, whether or not an individual has hypoglycemia or hypercalcemia is not determined by reference to insulin (14) or parathyroid hormone levels (15), respectively. Adrenocorticotropic hormone (ACTH) levels, though helpful in diagnosing adrenal autonomy, are not considered diagnostic for Cushing's syndrome (16). In general, the level of a controlling hormone is used to determine the cause of a disturbance rather than identifying whether or not there is a disturbance (14–16).

We, therefore, aimed at determining whether or not a systematic review of the literature might indicate the relative merits of thyroid hormone levels and TSH levels, in terms of associations with a broad range of clinical parameters. Because of the strong negative population correlation between free thyroxine (fT4) and TSH (17,18), we expected to find associations between both TSH and fT4 levels and the clinical features of thyroid dysfunction. We further reasoned that if the clinical features were associated better with TSH levels, the current rationale for thyroid function testing and the current consequent clinical and research classifications and practices would be supported, but, if the clinical features were associated better with thyroid hormone levels, these classifications and practices would warrant review. In this latter circumstance, the previously noted associations of clinical features with TSH levels could be attributed to the aforementioned strong negative population correlation between fT4 and TSH.

## Methods

### Search strategy

Up to October 9, 2019, a systematic search was performed of PubMed/MEDLINE by using the following terms: thyroxine (T4), fT4, total triiodothyronine (TT3), free triiodothyronine (fT3), TSH, and subclinical. No restrictions were placed on language, country, or publication date. The resulting literature was first examined to confirm the previously reported general trends of association between clinical parameters and thyroid status.

On account of the results of this first examination of the literature (see Results section), we studied atrial fibrillation (AF) and other cardiac parameters, bone density and fracture, cancer, death, frailty, dementia and associated pathology, obesity, features of the metabolic syndrome, and pregnancy outcomes. We specifically sought studies that addressed the associations between both TSH and thyroid hormone levels, determined simultaneously in the same individuals, with any of the clinical parameters just mentioned.

### Study selection and data extraction

Initially, the titles of the articles were screened for relevance and then the abstracts, with full-text reports of potentially relevant reports were reviewed. Additional relevant articles were searched for in the reference lists of the retrieved full-text studies. If repeated study was made of the same cohort, only the latest was included. The literature search data extraction, identification of additional relevant articles, and critical appraisal were conducted independently by two of the authors (S.P.F. and H.F.), and any discrepancies were resolved by consensus with reference to the criteria described in the next section. Should consensus regarding any article not have been achieved, the default position was that the article would be included. No study that contradicted the results of our work was knowingly excluded.

Studies reporting on associations of levels of fT4, TT3/fT3, and TSH with clinical features related to thyroid dysfunction were included. We included both TT3 and fT3, as there were relatively few studies of fT3. We also included analyses comparing associations with subclinical hypothyroidism and euthyroid hypothyroxinemia, reasoning that this is a comparison of low thyroid function defined on the basis of TSH levels or thyroid hormone levels, respectively. Reports were excluded if the studied population was <100 individuals. Review articles, editorials, meta-analyses, and meeting abstracts were also excluded.

The following information was extracted from each such study: first author, country, number of individuals, sex, age intervals, nature of the study, and the relevant clinical parameter. As there were many subtle different parameters examined, we also grouped the parameters into eight major phenotypes or systems: “cardiac,” bone,” “dementia,” “cancer,” “mortality,” “frailty,” “metabolic,” and “pregnancy.” We recorded any associations with thyroid hormones and/or TSH, in addition to the statistical techniques and degrees of significance of any associations (*p*-values and/or confidence limits). We also recorded the presence of “incongruent” associations, that is, associations in the opposite direction to that normally expected (e.g., obesity having associations with high thyroid function), or associations of thyroid hormones in the same direction as associations with TSH, as indicators of reverse causation (Supplementary Table S1) (19).

As our study was not directed at a collection of works addressing therapeutic outcomes of an intervention, the use of a quality assessment (the Newcastle–Ottawa scale) was adjusted to suit this setting. Principally, this adjustment consisted of allowing for continuous, as well as binary quantifications, of clinical outcomes and exposure to thyroid hormone levels. Articles were scored according to the representativeness of the subjects, the similarity of the subjects apart from differences in the parameter of interest, the reliability of the classification of thyroid status and parameter status, control for confounding factors, and for prospective studies, the demonstration that outcome was not present at study onset, the adequacy of length and completeness of follow-up. The Preferred Reporting Items for Systematic Reviews and Meta-analyses guidelines were followed (20).

### Statistical analysis

To determine whether thyroid hormone levels or TSH levels were associated better with the examined clinical parameters, we analyzed the earlier studies as to the relative frequencies of significant associations of thyroid hormone and TSH levels with the clinical parameters. We then performed further analyses to confirm that these findings did not result from any systematic bias.

We classified each result in a study as showing a significant result or a non-significant result. By a significant result, we mean that a given thyroid test has been shown to be associated with a given condition at a 5% significance level. We treated the result as a binary response variable with the levels of success (significant) and failure (non-significant). We combined TT3 and fT3 as TT3/fT3.

The predictors considered were the type of thyroid test (i.e., TSH, fT4, or TT3/fT3), the clinical system under consideration; the number of subjects in the analysis; and the number of covariates in the model. To account for the repeated analysis within each study, we also incorporated a random intercept term. We considered random intercepts for the study, the cohorts nested within each study, the type of analysis nested within the study, and the complexity of the models nested within the studies.

Pairwise comparisons of the thyroid tests were performed at a 5% overall significance level for those models where a significant effect of the predictor, type of thyroid test, was found. We calculated the Tukey pairwise comparisons between the thyroid tests by using the multcomp package (21). We conducted a McNemar analysis on the contingency tables for each comparative pair of thyroid tests. We tested the null hypothesis that there was no change in the proportion of significant results between the two thyroid tests under consideration. As a final attempt to account for dependency within each study, we performed a simple logistic regression analysis by using only a single randomly chosen analysis from the series of nested models in each study. We performed this for each of the following strata:

- smallest number of subjects, simple model;
- smallest number of subjects, complex model;
- largest number of subjects, simple model; and
- largest number of subjects, complex model.

We performed a sensitivity study minimizing the contribution of possible reverse causation, analyzing only the prospective analyses from studies that were free of incongruent associations.

All modeling was performed by using the lme4 (22) and lmerTest (23) packages in R (24), and all codes are available at https://github.com/jonotuke/TSH_2019.

## Results

We found, in our first examination of the literature, that though the findings were not unanimous, there was general consistency of the data. In general, consistent with prior work (8), AF (25–31), osteoporosis (32–39), and cancer (40–43) were associated with higher thyroid function that was defined by using TSH and/or thyroid hormone levels, across and beyond the reference range, and steatohepatitis (44–46) and other features of the metabolic syndrome (19,47–66) were associated with lower thyroid function. Both high and low thyroid function, as compared with mid-range thyroid function, were associated with clinical and pathological features of cognitive decline (26,67–75), frailty (76–79), total/cardiovascular mortality (26,80–88), cardiac physiology (89), cardiac disease (apart from AF) (26,31,67,83–85,88,90,91), and pregnancy outcomes (92–99).

There were many series finding these associations in the context of subclinical thyroid dysfunction. Many of these studies (25,50,51,67,83–85,87–89), however, did not address the relative associations of clinical parameters with TSH and thyroid hormone levels, the focus of our study.

**FIG. 1.**
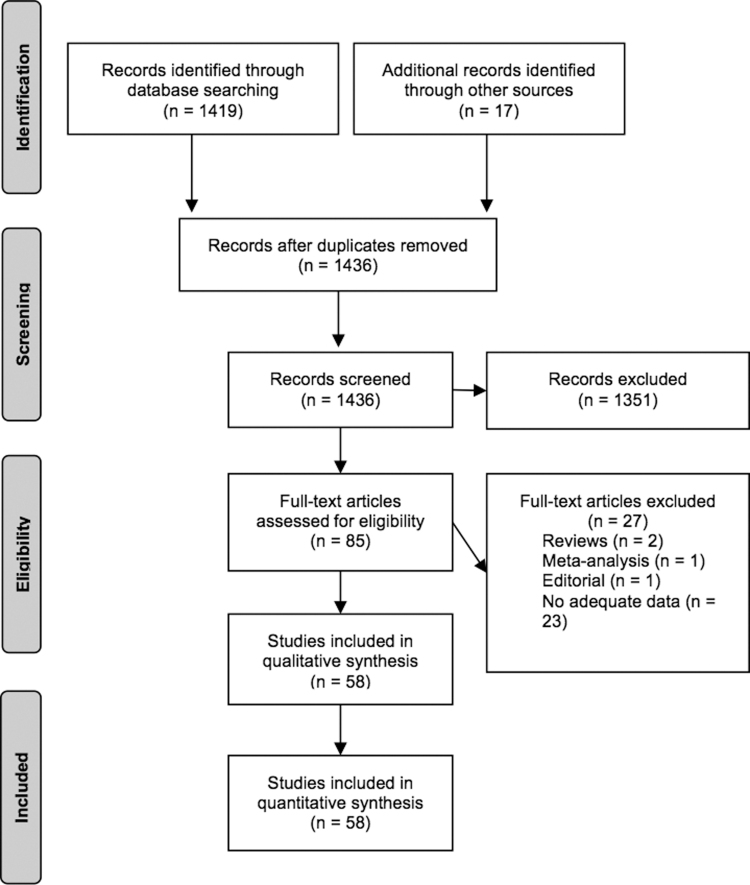
Description of literature search.

In the end, we identified 58 studies that addressed this question (Fig. 1; Table 1). We found no previous synthesis of the data on the effect of thyroid function, as measured by TSH in comparison to thyroid hormone levels, across a range of organ systems. One meta-analysis restricted to AF (27) was not included in our analysis. Many of the studies addressed multiple parameters summarized by those indicated in Table 1.

**Table 1.** Description and Quality Assessment of Included Studies

Study	Parameter	Cohort study	Population	N (% female)	Age	NOS
Gammage *et al.* (28)	AF	Cross-section	U.K. community	5860 (51)	72 (65–98)	9/9
Cappola *et al.* (26)	Multiple parameters	Prospective	U.S. community	2843 (56)	75 ± 5	9/9
Heeringa *et al.* (29)	AF	Cross-section	Netherlands community	1455 (59)	68 ± 8	9/9
Chaker *et al.* (30)	AF	Prospective	Netherlands community	9166 (57)	65 ± 9.9	9/9
Chaker *et al.* (86)	Sudden cardiac death	Prospective	Netherlands community age ≥45 years	10,318 (57)	65 ± 10	9/9
Kannan *et al.* (31)	Heart failure, cardiac outcomes	Cross-section	U.S. heart failure cohort	1365 (35)	56.6 ± 14.5	9/9
Peixoto de Miranda *et al.* (91)	Coronary artery disease	Cross-section	Brazil civil servants	767 (49.3)	58	9/9
Roef *et al*. (89)	Heart physiology	Cross-section	Belgium community	2078 (49)	M 46 (41–51)	9/9
					F 45 (40–50)	
van de Ven *et al.* (81)	Mortality	Prospective	Netherlands community	5816 (53)	56 ± 18	9/9
Inoue *et al.* (82)	Mortality	Prospective	U.S. community	5257 (not stated)	46 ± 17	8/9
Yeap *et al.* (80)	Mortality	Prospective	Australian community men	3885 (0)	77 ± 3	9/9
Chan *et al.* (40)	Cancer	Prospective	Australian community	3649 (56)	51 ± 15	8/9
Tosovic *et al.* (41)	Cancer	Prospective	Sweden community, women born during 1932–1950	17,035 (100)	56.6 ± 7.1^*^	9/9
Khan *et al.* (42)	Cancer	Prospective	Netherlands community	10,318 (57)	61 (57–68)	8/9
Kuijpens *et al.* (43)	Breast cancer	Cross-section and prospective	Netherlands community	2775 (100)	47–54	8/9
van den Beld *et al.* (78)	Frailty	Prospective + cross-section	Netherlands community men age ≥73 years	403 (0)	78 (73–94)	9/9
Yeap *et al.* (76)	Frailty	Cross-section	Australia community men	3943 (0)	75 ± 4	8/9
Bano *et al.* (77)	Frailty	Prospective	Netherlands community	9419 (57)	65 ± 10	9/9
			*n* = 9419; age 65 ± 10; female 57%			
Gussekloo *et al.* (79)	Frailty	Prospective	Netherlands community; all age 85	558 (66)	85	8/9
Volpato *et al.* (69)	Dementia	Prospective	U.S. community women age ≥65 years	464 (100)	77 ± 0.6	8/9
de Jong *et al.* (71)	Dementia	Cross-section	Netherlands community	489 (48)	73 ± 8	9/9
Chaker *et al.* (74)	Dementia	Prospective	Netherlands community	9446 (43.3)	64.9	9/9
Itterman *et al.* (75)	Dementia	Cross-section	Germany community	2557 (55)	51 ± 10^*^	9/9
Yeap *et al.* (73)	Dementia	Prospective	Australia community men	3401 (0)	79.2 ± 3.5^*^	8/9
Roef *et al.* (34)	Osteoporosis	Cross-section	Belgian community, men age 25–45 years	677 (0)	34 ± 6	9/9
van der Deure *et al.* (36)	Osteoporosis	Cross-section	Netherlands community age ≥55 years	1151 (58)	69 ± 8	9/9
Murphy *et al.* (35)	Osteoporosis	Cross-section	European post-menopausal women	1278 (100)	68 ± 7	7/9
van Rijn *et al.* (33)	Osteoporosis	Cross-section	Netherlands post-menopausal women	1477 (100)	50 ± 2	9/9
Lambrinoudaki *et al.* (39)	Vertebral fracture	Cross-section	Greece menopause clinic	335 (100)	56 ± 7.1^*^	9/9
Siru *et al.* (38)	Hip fracture, bone turnover	Prospective	Australian community men	338 (0)	76.7 ± 3.5	9/9
Waring *et al.* (37)	Bone loss, fracture	Prospective	U.S. community men	1602 (0)	73.6 ± 5.9^*^	9/9
Proces *et al.* (60)	Obesity	Cross-section	Belgium hospital outpatients	125 (44)	57 (13–89)	7/9
Wolide *et al.* (61)	Obesity					
Makepeace *et al.* (53)	Obesity/Met S	Cross-section	Australian community	1853 (47)	49 ± 17	9/9
			*n* = 1853; age 49 ± 17; female 47%			
Mehran *et al.* (47)	Obesity/Met S	Prospective	Iran community	2393 (61)	38 ± 13	9/9
Shon *et al.* (52)	Obesity/Met S	Cross-section	Korea women medical centre primary health screening	1572 (100)	46 ± 11	9/9
Roos *et al.* (48)	Obesity/Met S	Cross-section	Netherlands community	1581 (46)	48 ± 12	9/9
Jun *et al.* (55)	Obesity/Met S	Cross-section	Korea medical centre attendees	6235 (42)	50 ± 8	9/9
Xu *et al.* (45)	Obesity/Met S	Cross-section	China community	878 (37)	72 ± 4	9/9
Bano *et al.* (46)	Obesity/Met S	Prospective	Netherlands community	9640 (57)	65 ± 10	9/9
Ittermann *et al.* (44)	Obesity/Met S	Cross-section	Germany community	3661 (48)	M 51 ± 16	9/9
					F 48 ± 16	
Knudsen *et al.* (49)	Obesity/Met S	Cross section	Denmark community	4082 (“preponderance”)	18–65	9/9
Oh *et al.* (56)	Obesity/Met S	Cross-section	Korea community	4275 (50)	49	9/9
Garduño-Garcia *et al.* (49)	Obesity/Met S	Cross-section	Mexico community	3033 (51)	42 ± 10	9/9
Temizkan *et al.* (62)	Met S	Cross-section	Turkey obesity clinic	1275 (83)	38 ± 11	9/9
Jain *et al.* (63)	Lipids	Cross-section	U.S. population	3862 (not stated)	Not stated	9/9
Boekholdt *et al.* (64)	Met S	Cross-section	U.K. community	11,554 (54)	58 ± 9	9/9
Udenze *et al.* (65)	Met S	Cross-section	Nigeria university staff	150 (54)	46.3 ± 8.1	8/9
Elgazar *et al.* (66)	Diabetes	Cross-section	Egypt hospital outpatients	400 (61)	54 ± 4.9^*^	7/9
Chaker *et al.* (54)	Diabetes	Prospective	Netherlands community	8542 (58)	65 ± 10	9/9
Vrijkotte *et al.* (92)	Foetal growth	Prospective	Netherlands community	3988 (100)	31 ± 4.8	9/9
Korevar *et al.* (93)	Premature delivery	Prospective	Netherlands community	5971 (100)	29.7 ± 5.0	9/9
Medici *et al.* (94)	Hypertensive pregnancy	Prospective	Netherlands community	5153 (100)	29.7 ± 5.1	9/9
Cleary-Goldman *et al.* (95)	Pregnancy outcome	Prospective	U.S. community	10,990 (100)	29.6 ± 5.6	9/9
Breathnach *et al.* (96)	Placental abruption	Prospective	Ireland community	904 (100)	26 ± 6^*^	9/9
Ashoor *et al.* (97)	Foetal death	Retrospective	England hospital	3794 (100)	32.2 (28–36)	8/9
Knight *et al.* (98)	Pregnancy-metabolic parameters	Cross-section	England community	741 (100)	30.1 ± 5.1	9/9
Li *et al.* (99)	Pregnancy-childhood development	Prospective	China clinic	1268 (100)	28 ± 2	9/9

^*^ Age of largest subset of population.

F, female; M, male; Met S, metabolic syndrome; NOS, adapted Newcastle–Ottawa quality assessment scale (the higher number out of 9, the better the study).

We found 22 studies (19,26,30,31,34,35,39,41,44,45,48,55,60–62,65,66,71,75,78,79,91) that examined associations with fT4, TT3/fT3, and TSH and a further 36 studies (26,29,33,36–38,40,42,43,45,47,49,52–54,56,63,64,69,73,74,76,77,80–82,86,90,92–99) that examined associations with only fT4 and TSH levels.

These 58 studies included cross-sectional and prospective cohort studies, diverse populations, and both sexes. They were contemporary and of high quality (Table 1). The study populations comprised strictly euthyroid subjects (26,29,30,34,39,45,48,52–55,62,69,81,82,86,90,91), subjects either euthyroid or with subclinical thyroid dysfunction (19,33,35,36,38,40,42,47,49,60,65,71,73,75–78,80,92,93,95,96,98,99), and subjects euthyroid or with subclinical/overt thyroid dysfunction (28,31,37,41,43,44,46,47,56,61,63,64,66,74,79,94). In some studies, different subsets were examined separately. The 58 articles included in our meta-analysis yielded 1880 results of associations analysis. The supplement catalogues all of these associations in terms of clinical parameters, subgroups, number of participants, statistical methods, statistical significance, and *p*-values/confidence limits.

The number of subjects for each analysis ranged from 18 to 10,990 with a mean of 3071 (median 2078). The number of results in each study ranged from 3 (60) to 180 (92).

**FIG. 2.**
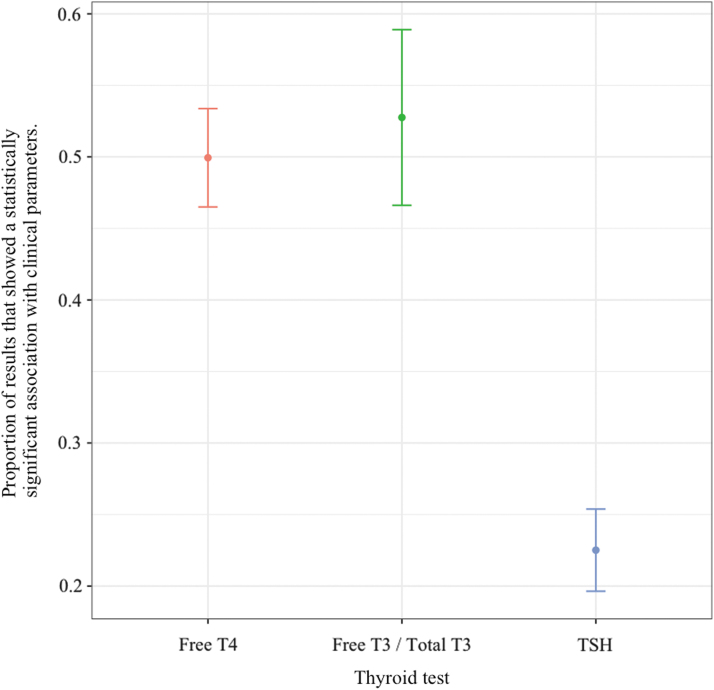
Overall associations of thyroid hormone and TSH levels with clinical parameters. T3, triiodothyronine; T4, thyroxine; TSH, thyrotropin.

Analysis of all these data confirmed the superiority of associations with thyroid hormone levels (fT4, TT3/fT3) as compared with TSH levels (Fig. 2). fT4 had a significant association with a clinical parameter in 50% of the analyses of the articles. TT3/fT3 had a significant association in 53% of the analyses, whereas TSH had a significant association in only 23%. fT4 levels were associated with clinical parameters and were statistically significantly more often than with TSH levels, (*p* < 0.0001), as did TT3/fT3, (*p* < 0.0001). The difference between fT4 and TT3/fT3 levels was not significant (*p* = 0.71).

When there was a significant association with fT4, the association with TSH was simultaneously significant 30% of the time; for the converse, the frequency was 66%. For TT3/fT3, the respective figures were similar at 33% and 62%. The McNemar analysis demonstrated these results to be significant; for the comparisons of fT4 versus TSH and TT3/fT3 versus TSH, the null hypothesis was rejected (*p* < 0.0001). For the comparison of fT4 versus TT3/fT3, we failed to reject the null hypothesis (*p* < 0.4305).

**FIG. 3.**
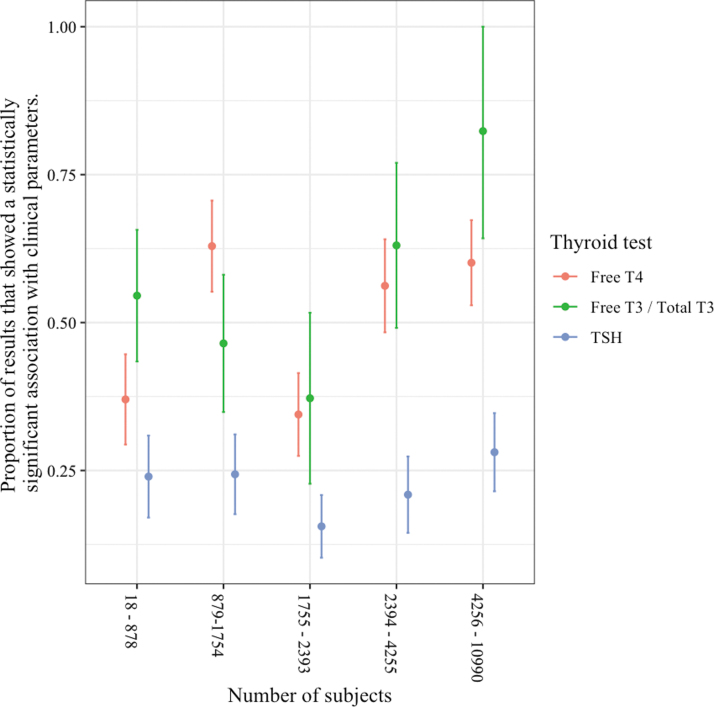
Associations of thyroid hormone and TSH levels with clinical parameters according to sample size.

**FIG. 4.**
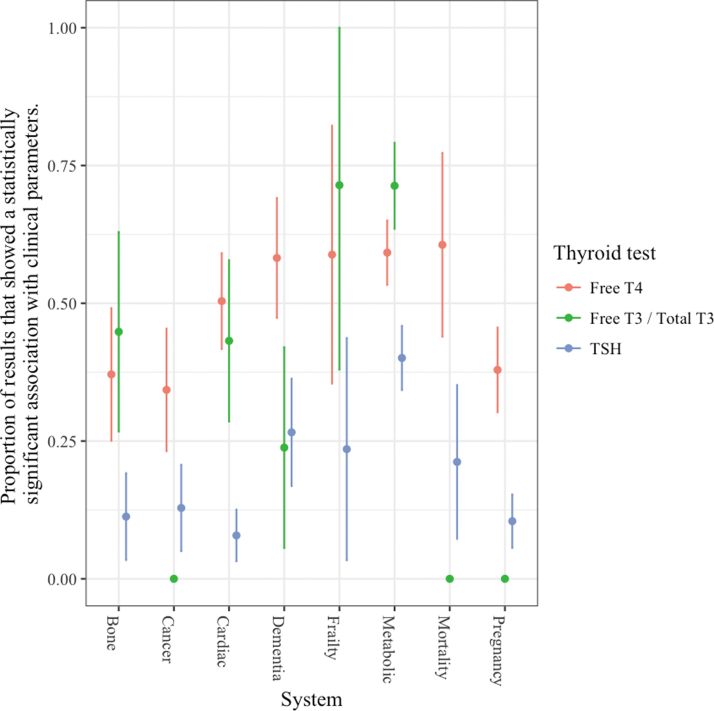
Associations of thyroid hormone and TSH levels with clinical parameters according to clinical system.

**FIG. 5.**
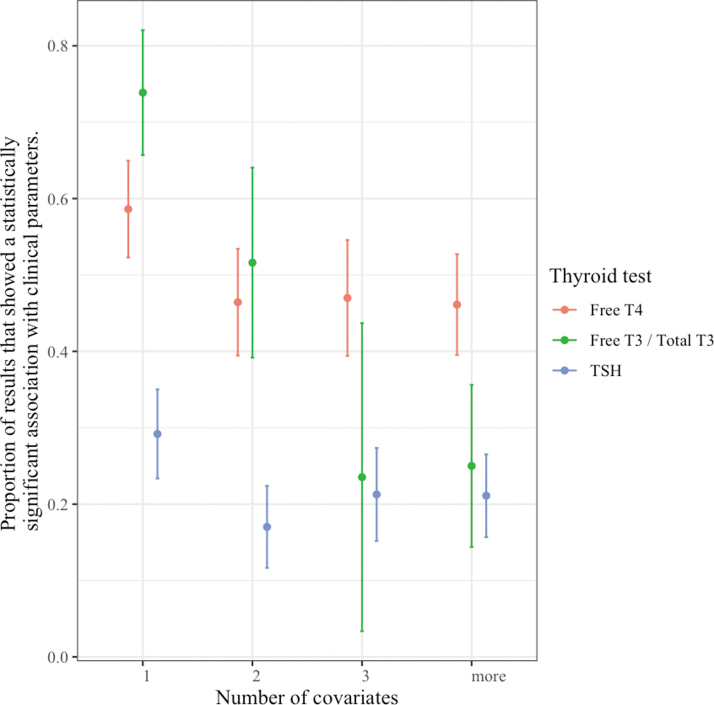
Associations of thyroid hormone and TSH levels with clinical parameters according to number of covariates.

As the number of subjects in the analysis increased, the superior associations with thyroid hormones did not diminish (Fig. 3), and similarly the system did not play a significant role (Fig. 4). In an analysis including the number of covariates in the original result, at higher numbers of covariates, the association of clinical parameters with TT3/fT3 levels was no longer significantly different from the association with TSH levels (Fig. 5).

The basic analysis provided earlier ignores the many sources of dependence between the results reported in each study. To account for this, it was necessary to incorporate a random intercept for study in the model (*p* = 2.2 × 10^−16^). There was still then a statistically significant effect of thyroid test in predicting the significance of results (*p* < 2.2 × 10^−16^). *Post hoc* pairwise comparisons show that there was a statistically higher proportion of significant results for fT4 compared with TSH (*p* < 1 × 10^−5^), and also a statistically higher proportion of significant results for TT3/fT3 compared with TSH (*p* < 1 × 10^−5^). These results confirmed those illustrated in the earlier mentioned confidence interval plots. We found that the additional main effects of system, cohort size, and number of covariates again did not improve the predictive effect of the model, compared with one with just a thyroid test (based on minimizing the Bayesian Information Criterion). We found that a nested random effects structure of cohort within study was a statistically valid addition to the model, but it did not change the observed effects of the thyroid test on what has been cited earlier.

We found, when addressing the issue of dependence of results, a statistically significant effect of thyroid test on the proportion of statistically significant results. Pairwise comparisons revealed that the only significant results in all four models were for fT4 having more significant associations than TSH (Table 2). In this analysis, TT3/fT3 levels did not have more associations with clinical parameters than levels of TSH.

**Table 2.** Model Description

	Thyroid test	fT4 vs. TSH
Smallest number of subjects, simple model	*p* = 0.0001891	*p* = 0.000191
Smallest number of subjects, complex model	*p* = 0.000773	*p* = 0.000658
Largest number of subjects, simple model	*p* = 0.01126	*p* = 0.00931
Largest number of subjects, complex model	*p* = 0.0006587	*p* = 0.000696

fT4, free thyroxine; TSH, thyrotropin.

The results of our sensitivity analysis aimed at minimizing any effect of reverse causation showed no significant change in the proportion of fT4 and TSH levels being associated with clinical parameters, and hence in the statistical conclusions. However, the association of TT3/fT3 levels with clinical parameters was not significantly different than with TSH levels. The proportion of associations with TT3/fT3 was only 13% in this analysis as compared with 53% in the full analysis.

There was no significant change to any of our results regarding the TT3/fT3 combination with consideration of TT3 and fT3 separately.

Only a few of the studies included patients on T4 therapy. In these studies, the proportion of patients on T4 was very low such that separate analyses of these patients were not undertaken. Analyses of cohorts with removal of these patients did not affect the results.

## Discussion

We believe this is the first systematic review studying TSH and thyroid hormone associations with various clinical parameters. The results indicate that, contrary to the current paradigm, thyroid hormone levels are associated more strongly with clinical parameters than TSH levels. Any relationship of clinical parameters with TSH levels can be explained by the strong population relationship between thyroid hormone levels and TSH levels, such that TSH levels are merely indirect measures of thyroid hormone levels.

In our sample, we found no indication of, or reference to, any work that suggested that TSH levels consistently indicate thyroid status of any organ or tissue more strongly than thyroid hormone levels.

As our goal was not to estimate the effect size for one treatment, our meta-analysis methodology differed from some other meta-analysis methodologies in that we did not use a weighted technique or pool all original patient data. In addition, it would not have been appropriate to combine all of these factors by using such meta-analysis methodology, as our analysis encompassed multiple studies covering various clinical outcomes, using different methodologies, different assays, and statistical methods.

Theoretically, one could use such other methodology to do a meta-analysis of each clinical parameter, but these individual meta-analyses would still need to be combined by using a method akin to ours (i.e., summing the meta-analyses in some way) to determine whether levels of thyroid hormones or TSH are more likely to be associated in general with clinical parameters. Further, in using such a technique of analysis, the information from many of the studies in our sample would be lost as the parameter/population/statistical method might not be amenable to pooling (27).

The results of the individual patient meta-analysis of AF (27) do, in fact, support our conclusions, showing superior associations with fT4 levels than with TSH levels. Also supportive is a recently published similar meta-analysis of pre-term delivery (100), showing that fT4 levels are associated with the clinical parameter at least as well as TSH levels. One could even argue that the results of these two conventional meta-analyses alone disprove the general hypothesis that TSH levels provide a better guide to thyroid status than fT4 levels.

Potentially, the summation of statistically significant results can be unreliable (20], but we have accounted for the possibilities of bias on account of imbalance in the size of the studies, the nature of the parameters, and the possibility of reverse causation. In all of the studies (except for the few studies comparing individuals with subclinical hypothyroidism with individuals with isolated hypothyroxinemia), each subject was his/her own control, and the study populations of many of the studies were unselected members of a community, so the risk of bias from these considerations was obviated. The convincing degree of superiority of thyroid hormone levels as compared with TSH levels also provides a buffer against the possibility of some unidentified bias influencing our results.

The strictest interpretation of our data would, nevertheless, qualify our conclusions such that they would be valid only to the degree that the chosen parameters truly reflect the thyroid state. Residual confounding, by mechanisms as yet incompletely understood, may have affected the reported associations between thyroid function tests and all of the clinical parameters. This possibility, not considered to be likely in the studies we reviewed, would also compromise the previous literature describing the considered consequences of sub-clinical thyroid dysfunction.

However, even in these circumstances, a slightly weaker conclusion for our study—that is, that there is no evidence supporting the superiority of TSH levels in the assessment of thyroid function—would still stand. Further, there is much evidence to indicate that at least some of the chosen parameters do truly reflect the thyroid state.

In particular, although there are clinical parameters that can affect thyroid function, we do not believe that such reverse causation significantly influence our results. Reverse causation mechanisms have been described for parameters associated with low thyroid function (e.g., obesity (101–103) and dyslipidemia (57,58,104)), but in these circumstances the reverse causation effects would tend to lead to greater associations with TSH levels rather than with fT4 levels.

The sensitivity of TT3/fT3 levels to the sick euthyroid state (105), generated by altered deiodinase activity (106), may also explain some of the associations with TT3/fT3. In particular, mortality and frailty may be associated with low TT3/fT3 levels via reverse causation. As the TSH would also be expected to be low in this situation, one might expect incongruent associations between clinical parameters, and TT3/fT3 (and possibly fT4) and TSH. Our sensitivity study excluded such studies.

We are not aware of any association of a clinical parameter with a high fT4 having been linked to reverse causation. If anything, any component of the sick euthyroid state associated with these conditions, by lowering TSH and fT4 (105), should again favor an association with TSH rather than fT4.

Mendelian randomization studies have provided evidence that the relationship between thyroid function and AF is causal (107,108), whereas there may be reverse causation underlying the relationship between thyroid function and obesity (109). Other indicators supporting a causative relationship between thyroid function and at least some of the parameters we examined include the relationships being seen in otherwise healthy individuals (91–99), the prospective nature of many of our included studies, our sensitivity study, the observed similarity of the relationships to those seen in overt thyroid disease (110–120), basic science evidence (121,122), and positive animal (123) and human (19) intervention studies.

Nevertheless, additional intervention studies could provide further evidence as to the direction of causality in the associations we have studied. Ideally, such intervention studies would be designed to ensure that the intervention, rather than merely normalizing TSH levels, significantly changes the levels of thyroid hormones.

We found TT3/fT3 level associations with fewer parameters than we found for fT4. Although TT3/fT3 levels were associated more strongly than TSH levels, and equally strongly as fT4 levels, with clinical parameters, our sensitivity study showed a fall in the frequency of TT3/fT3 associations, suggesting a component of reverse causation.

Two other analyses, the analysis of associations according to the number of covariates and the sampling analysis, also indicated that the associations of clinical parameters with TT3/fT3 may not be as robust as the associations with fT4 levels. Overall, TT3/fT3 measurement added little to the assessment based on fT4 levels. Future studies may further clarify the relative importance of fT4 and fT3 levels.

fT4 is not the active thyroid hormone at the cellular nuclear level (106). The strong relationships of parameters, especially AF (risk increased up to 9 × across the normal reference range (30)), with levels of fT4 indicate that the active intracellular triiodothyronine generated by thyroid hormone transporters and deiodinases (106) appears to be, at least in the heart, proportional to circulating fT4. Any discrepancy, indicating local regulation of thyroid effect, may be more prominent in more severe pathophysiological circumstances (106), and therefore more relevant in the circumstances of multisystem entities such as frailty, death, and metabolic disturbance.

Our results do not imply that no information can be gleaned from the presence of an abnormal TSH level. In the presence of normal thyroid hormone levels, such TSH levels indicate that the thyroid gland physiology is abnormal. However, for the function of other tissues and organs, the TSH level required to maintain a given level of thyroid hormones appears generally not to be relevant.

It remains possible too that additional analyses might find that TSH levels are providing an additional signal to fT4 levels, in some populations for some conditions. It has been suggested that TSH itself may have physiological effects apart from the stimulation of thyroid hormone levels (36,124), and such effects rather than via the reflection of thyroid status might explain such a TSH signal. Empirically, thus far, the evidence suggests that any of these TSH effects are small.

The association of thyroid hormone and particularly fT4 levels, rather than TSH levels, with clinical features has been noted by many authors, covering many individual parameters (26–28,30,33,35,42,44,46–48,52,53,57,69,73,76,80,81,86). In particular, the meta-analysis regarding AF noted the association with fT4 but not with TSH (27). Authors also previously found evidence of associations of clinical parameters with fT4 in the absence of an association with subclinical thyroid dysfunction as currently diagnosed (33,49,73,80,86). One of these studies also showed associations with TSH (49).

Nevertheless, to date, to the best of our knowledge, this information from the individual studies showing the superiority of thyroid hormone levels in terms of associations with individual clinical parameters has not been synthesized into a formal conclusion regarding the biochemical assessment of thyroid function in general.

It has been suggested that despite TSH being considered a more sensitive indicator of thyroid status, fT4 may be a more sensitive indicator of “cardiac” (28), or “tissue” (47,53) thyroid status. Our study strengthens and generalizes these propositions, indicating that fT4 is the more sensitive indicator of thyroid status because it is the better indicator of tissue and organ effects.

The superior association of clinical parameters with fT4 as compared with TSH levels has more often been attributed to a putative disturbance of set point physiology (42,46,47,69,76,81,86), to a significant difference between pituitary and peripheral sensitivity to fT4 (27,46,48,52), or to statistical/other factors (including reverse causation) (33,36,44,49,58).

The explanations related to set points are denied in the first instance by the evidence that the relatively stable thyroid hormone levels seen in individuals are better explained by a model of “balance points” (or “equilibrium points”) rather than “set points” (125). Notwithstanding this concept, it has been suggested that in older adults there is an alteration of what is termed “set point physiology,” in that TSH may be less suppressed by any given rise in fT4 (42,76). However, in this situation, though the range of TSH may change, any physiological association with greater or lesser TSH levels should remain intact. Further, the greater association of clinical parameters with fT4 rather than TSH levels is apparent across a wide age range (Table 1).

At a population level, TSH levels do, indeed, decrease with rising fT4 levels (17,18), suggesting that in general pituitary sensitivity to thyroid hormones is robust. If for any reason there were a disturbance to pituitary sensitivity in the absence of a corresponding change to peripheral sensitivity, this would in any event provide another reason not to diagnose thyroid function on the basis of TSH levels.

The evidence also suggests that, regardless of the method used, the classification of thyroid function into normal, subclinical disease and overt disease is arbitrary. Thyroid hormones, as previously suggested (9,26), similar to many other biological parameters, exert a continuum of effects across the normal range. There is no clear border between normal and abnormal. There are advantages and disadvantages associated with all levels (9,26,126). Individuals with relatively low levels of fT4, for example, are less likely to develop AF but more likely to develop metabolic syndrome; the converse applies for individuals with higher fT4 levels. At the extremes, the disadvantages clearly outweigh the advantages, and individuals are likely to become symptomatic.

On the other hand, any excursion from the middle of the range has an association with some pathology or other. Some individual pathologies, for example, frailty, mortality, and dementia may increase with deviations either side of the middle of the range. It seems likely that evolutionary mechanisms have arisen to minimize variation from the middle of the reference range of thyroid hormones (127).

The fact that TSH levels reliably identify overt thyroid dysfunction can also be explained by the negative population relationship between TSH and fT4, that is, its extension into the abnormal ranges of fT4 (17,18). This extension is due merely to the fact that the vast majority of all overt thyroid dysfunction is primary rather than secondary (128). This situation differs from other endocrine pathology, for example Cushing's syndrome, where ACTH levels cannot be used as a screening test on account of the likelihood that Cushing's syndrome may be secondary, that is, be due to a disorder of ACTH regulation (129). The fact that TSH levels are thereby very sensitive screening tests for overt thyroid dysfunction (130) does not imply that TSH levels are very specific, that is, that an abnormal TSH level implies thyroid dysfunction. Our work indicates that an abnormal TSH level *per se* is an imprecise indicator of tissue or organ hyper/hypothyroidism as compared with thyroid hormone levels.

This work addressed diagnosis alone. Extrapolation of our findings appears logical, and there is no apparent *a priori* reason as to why TSH levels should be preferred over thyroid hormone levels in the context of monitoring thyroid treatments. Randomized trials might, nevertheless, reveal that additional considerations apply in these circumstances. Though there was no suggestion in the studies that we examined of a difference with individuals on thyroid hormone replacement, their numbers were small.

In summary, there is now matching theoretical and empiric evidence from a variety of sources suggesting that the thyroid status of an individual is better defined by thyroid hormone levels than TSH levels. There is evidence of a continuum of thyroid hormone effects along the continuum of thyroid hormone levels, with a possible optimum around the middle of the reference range. Though TSH levels remain good screening tests for overt thyroid dysfunction, it is theoretically and empirically more sound to rely on thyroid hormone and especially fT4 levels to classify the thyroid state.

This work should result in a simplification of the understanding of thyroid physiology and pathophysiology, and bring it more into line with the understanding of the physiology and pathophysiology of other parameters, whereby the status of a parameter is judged by its level rather than the level of any controlling factor. Reconsideration of the TSH-based diagnostic approach to thyroid function appears to be indicated. In turn, this would appear to have implications for clinical guidelines, research methodology, and the rationale of underlying physiological principles.

## Supplementary Material

Supplemental data

## References

[1] Cappola AR, Desai AS, Medici M, Cooper LS, Egan D, Sopko G, Fishman GI, Goldman S, Cooper DS, Mora A, Kudenchuk PJ, Hollenberg AN, McDonald CL, Ladenson PW 2019 Thyroid and cardiovascular disease: research agenda for enhancing knowledge, prevention and treatment. Thyroid 29:760–777 31081722 10.1089/thy.2018.0416 PMC6913785

[2] Schneider C, Feller M, Bauer DC, Collet T-H, da Costa BR, Auer R, Peeters RP, Brown SJ, Bremner AP, O'Leary PC, Feddema P, Leedman PJ, Aujesky D, Walsh JP, Rodondi N 2018 Initial evaluation of thyroid dysfunction—are simultaneous TSH and fT4 tests necessary? PLoS One 13:e0196631 29709030 10.1371/journal.pone.0196631 PMC5927436

[3] Orgiazzi J 2016 Dose normal TSH mean euthyroidism in L-T4 treatment? Clin Thyroidol 28:325–328

[4] Ross DS 2019 Euthyroid hyperthyroxinemia and hypothyroxinemia. Cooper DS (ed) Waltham, MA: UpToDate, Inc. Available at https://www.uptodate.com (accessed 1110, 2019)

[5] Wilson S, Parle JV, Roberts LM, Roalfe AK, Hobbs FDR, Clark P, Sheppard MC, Gammage MD Pattison HM, Franklyn JA 2006 Prevalence of subclinical thyroid dysfunction and its relation to socioeconomic deprivation in the elderly: a community-based cross-sectional survey. J Clin Endocrinol Metab 91:4809–4816 17003083 10.1210/jc.2006-1557

[6] Biondi B, Cooper DS 2018 Subclinical hyperthyroidism. N Engl J Med 378:2411–2419 29924956 10.1056/NEJMcp1709318

[7] Palacios SS, Pascual-Corrales E, Galofre JC 2012 Management of subclinical hyperthyroidism. Int J Endocrinol Metab 10:490–496 23843809 10.5812/ijem.3447 PMC3693616

[8] Fatourechi V 2009 Subclinical hypothyroidism: an update for primary care physicians. Mayo Clin Proc 84:65–71 19121255 10.4065/84.1.65 PMC2664572

[9] Taylor PN, Razvi S, Pearce SH, Dayan CM 2013 A review of the clinical consequences of variation in thyroid function within the reference range. J Clin Endocrinol Metab 98:3562–3571 23824418 10.1210/jc.2013-1315

[10] The TRUST Study Group 2017 Thyroid hormone therapy for older adults with subclinical hypothyroidism. N Engl J Med 376:2534–2544 28402245 10.1056/NEJMoa1603825

[11] Villar HC, Sacconato H, Valente O, Atallah AN 2007 Thyroid hormone for subclinical hypothyroidism. Cochrane Database Syst Rev 18:CD003419 10.1002/14651858.CD003419.pub2 PMC6610974 17636722

[12] Hoermann R, Larisch R, Dietrich JW, Midgley JEM 2016 Derivation of a multivariate reference range for pituitary thyrotropin and thyroid hormones: diagnostic efficiency compared with conventional single reference method. Eur J Endocrinol 174:735–743 26951601 10.1530/EJE-16-0031

[13] Ross HA, den Hejer M, Hermus Ad RMM, Sweep FCGC 2009 Composite reference interval for thyroid-stimulating hormone and free thyroxine, comparison with common cutoff values, and reconsideration of subclinical thyroid disease. Clin Chem 55:2019–2025 19713278 10.1373/clinchem.2009.124560

[14] Cryer PE, Davis SN 2015 Chapter 420: Hypoglycemia. In: Kasper DL, Hauser SL, Jameson JL, Fauci AS, Longo DL, Loscalzo J (eds) Harrison's Principles of Internal Medicine, 19th ed. McGraw Hill, New York, pp. 2430–2435

[15] Khosla S 2015 Chapter 65: Hypercalcemia and hypocalcemia. In: Kasper DL, Hauser SL, Jameson JL, Fauci AS, Longo DL, Loscalzo J (eds) Harrison's Principles of Internal Medicine, 19th ed. McGraw Hill, New York, pp, 313–314

[16] Arlt W 2015 Chapter 406: Disorders of the adrenal cortex. In: Kasper DL, Hauser SL, Jameson JL, Fauci AS, Longo DL, Loscalzo J (eds) Harrison's Principles of Internal Medicine, 19th ed. McGraw Hill, New York, pp. 2316

[17] Hoermann R, Eckl W, Hoermann C, Larisch R 2010 Complex relationship between free thyroxine and TSH in the regulation of thyroid function. Eur J Endocrinol 162:1123–1129 20299491 10.1530/EJE-10-0106

[18] Hadlow NC, Rothacker KM, Wardrop R, Brown SJ, Lim EU, Walsh JP 2013 The relationship between TSH and free T4 in a large population is complex and nonlinear and differs by age and sex. J Clin Endocrinol Metab 98:2936–2943 23671314 10.1210/jc.2012-4223

[19] Knudsen N, Laurberg P, Rasmussen LB, Bulow I, Perrild H, Ovesen L, Jørgensen T 2005 Small differences in thyroid function may be important for body mass index and the occurrence of obesity in the population. J Clin Endocrinol Metab 90:4019–4024 15870128 10.1210/jc.2004-2225

[20] Moher D, Liberati A, Tetzlaff J, Altman DG; PRISMA Group 2009 Preferred reporting items for systematic reviews and meta-analyses: the PRISMA statement. PLoS Med 6:e1000097 19621072 10.1371/journal.pmed.1000097 PMC2707599

[21] Hothorn T, Bretz F, Westfall P 2008 Simultaneous inference in general parametric models. Biom J 50:346–363 18481363 10.1002/bimj.200810425

[22] Bates D, Maechler M, Bolker B, Walker S 2015 Fitting linear mixed-effect models using lme4. J Stat Softw 67:1–48

[23] Kuznetsova A, Brockhoff PB, Christensen RHB 2017 lmerTest Package: tests in linear mixed effects models. J Stat Softw 82:1–26

[24] R Core Team. R: A language and environment for statistical computing. R Foundation for statistical computing, Vienna, Austria. Available at https://www.R-project.org (accessed 715, 2019)

[25] Selmer C, Olesen JB, Hansen ML, Lindharsen J, Olsen A-MS, Madsen JC, Hansen PR, Pedersen OD, Faber J, Torp-Pedersen C, Gislason GH 2012 The spectrum of thyroid disease and risk of new onset atrial fibrillation: a large population cohort study. BMJ 345:e7895 23186910 10.1136/bmj.e7895 PMC3508199

[26] Cappola AR, Arnold AM, Wulczn K, Carlson M, Robbins J, Psaty BM 2015 Thyroid function in the euthyroid range and adverse outcomes in older adults. J Clin Endocrinol Metab 100:1088–1096 25514105 10.1210/jc.2014-3586 PMC4333030

[27] Baumgartner C, da Costa BR, Collet TH, Feller M, Floriani C, Bauer DC, Cappola AR, Heckbert SR, Ceresini G, Gussekloo J, den Elzen WPJ, Peeters RP, Luben R, Völzke H, Dörr M, Walsh JP, Bremner A, Iacoviello M, Macfarlane P, Heeringa J, Stott DJ, Westendorp RGJ, Khaw KT, Magnani JW, Aujesky D, Rodondi N; Thyroid Studies Collaboration 2017 Thyroid Studies Collaboration. Thyroid function within the normal range, subclinical hypothyroidism, and the risk of atrial fibrillation. Circulation 136:2100–2116 29061566 10.1161/CIRCULATIONAHA.117.028753 PMC5705446

[28] Gammage MD, Parle JV, Holder RL, Roberts LM, Hobbs FD, Wilson S, Sheppard MC, Franklyn JA 2007 Association between serum free thyroxine concentration and atrial fibrillation. Arch Intern Med 167:928–934 17502534 10.1001/archinte.167.9.928

[29] Heeringa J, Hoogendoorn EH, van der Deure WM, Hofman A, Peeters RP, Hop WC, den Heijer M, Visser TJ, Witterman JC 2008 High-normal thyroid function and the risk of atrial fibrillation: the Rotterdam study. Arch Int Med 168:2219–2224 19001198 10.1001/archinte.168.20.2219

[30] Chaker L, Heeringa J, Deghan A, Medici M, Visser WE, Baumgartner C, Hofman A, Rodondi N, Peeters RP, Franco OH 2015 Normal thyroid function and the risk of atrial fibrillation: the Rotterdam study. J Clin Endocrinol Metab 100:3718–3724 26262438 10.1210/jc.2015-2480

[31] Kannan L, Shaw PA, Morley MP, Brandimarto J, Fang JC, Sweitzer NK, Cappola TP, Cappola AR 2018 Thyroid dysfunction in heart failure and cardiovascular outcomes. Circ Heart Fail 11:e005266 30562095 10.1161/CIRCHEARTFAILURE.118.005266 PMC6352308

[32] Yan Z, Huang H, Li J, Wang J 2016 Relationship between subclinical thyroid dysfunction and the risk of fracture: a meta-analysis of prospective cohort studies. Osteoporosis Int 1:115–125 10.1007/s00198-015-3221-z 26223189

[33] Van Rijn LE, Pop VJ, Williams GR 2014 Low bone mineral density is related to high physiological levels of free thyroxine in peri-menopausal women. Eur J Endocrinol 170:461–468 24336745 10.1530/EJE-13-0769

[34] Roef G, lapauw B, Goemaere S, Zmierczak H, Fliers T, Kaufman JM, Taes Y 2011 Thyroid hormone status within the physiological range affects bone mass and density in healthy men at the age of peak bone mass. Eur J Endocrinol 164:1027–1034 21393448 10.1530/EJE-10-1113

[35] Murphy E, Glüer CC, Reid DM, Felsenberg D, Roux C, Eastell R, Williams GR 2010 Thyroid function within the upper normal range is associated with reduced bone mineral density and an increased risk of nonvertebral fractures in healthy euthyroid postmenopausal women. J Clin Endocrinol Metab 95:3173–3181 20410228 10.1210/jc.2009-2630

[36] Van der Deure WM, Uitterlinden AG, Hofman A, Rivadeneira F, Pols HA, Peeters RP, Visser TJ 2008 Effects of serum TSH and FT4 levels and the TSHR-Asp727Glu polymorphism on bone: the Rotterdam study. Clin Endocrinol (Oxf) 68:175–181 17803697 10.1111/j.1365-2265.2007.03016.x

[37] Waring AC, Harrison S, Fink H, Samuels MH, Cawthorn PM, Zmuda JM, Orwoll ES, Bauer D 2013 A prospective study of thyroid function, bone loss, and fractures in older men: the MrOS study. J Bone Miner Res 28:472–479 23018684 10.1002/jbmr.1774 PMC4095773

[38] Siru R, Alfonso H, Chubb SAP, Golledge J, Flicker L, Yeap BB 2017 Subclinical thyroid dysfunction and circulating thyroid hormones are not associated with bone turnover markers or incident hip fracture in older men. Clin Endocrinol 89:93–99 10.1111/cen.13615 29655173

[39] Lambrinoudaki I, Armeni E, Pliatsika P, Rizos D, Kaparos G, Augoulea A, Alexandrou A, Flokatoula M, Creatsa M, Panoulis C, Triantafyllou N, Papacharalambous X 2017 Thyroid function and autoimmunity are associated with the risk of vertebral fractures in postmenopausal women. J Bone Miner Metab 35:227–233 27023333 10.1007/s00774-016-0752-0

[40] Chan YX, Knuiman MW, Divitini ML, Brown SJ, Walsh J, Yeap BB 2017 Lower TSH and higher free thyroxine predict incidence of prostate but not breast, colorectal or lung cancer. Eur J Endocrinol 177:297–308 28684452 10.1530/EJE-17-0197

[41] Tosovic A, Becker C, Bondeson A-G, Bondeson L, Ericsson U-B, Malm J, Manjer J 2012 Prospectively measured thyroid hormones and thyroid peroxidase antibodies in relation to breast cancer risk. Int J Cancer 131:226–2133 10.1002/ijc.27470 22323002

[42] Khan SR, Chaker L, Ruiter R, Aerts JGJV, Hoffman A, Deghan A, Franco OH, Stricker BHC, Peeters RP 2016 Thyroid function and cancer risk: the Rotterdam study. J Clin Endocrinol Metab 12:5030–5036 10.1210/jc.2016-2104 27648963

[43] Kuijpens JLP, Nykličtek I, Louwman MWJ, Weetman TAP, Pop VJM, Coebergh J-WW 2005 Hypothyroidism might be related to breast cancer in post-menopausal women. Thyroid 15:1253–1259 16356089 10.1089/thy.2005.15.1253

[44] Ittermann T, Haring R, Wallaschofski H, Baumeister S, Nauck M, Dörr M, Lerch M, Meyer zu Schwabedissen HE, Rosskopf D, Völzke H 2012 Inverse association between serum free thyroxine levels and hepatic steatosis: results from the study of health in Pomerania. Thyroid 22:568–574 22574630 10.1089/thy.2011.0279 PMC3358110

[45] Xu C, Xu L, Yu M, Li Y 2011 Association between thyroid function and non alcoholic fatty liver disease in euthyroid elderly Chinese. Clin Endocrinol 75:240–246 10.1111/j.1365-2265.2011.04016.x 21521285

[46] Bano A, Chaker L, Plompen EPC, Hofman A, Deghan A, Franco OH, Janssen HLA, Murad SW, Peeters RP 2016 Thyroid function and the risk of non-alcoholic fatty liver disease: the Rotterdam study. J Clin Endocrinol Metab 101:3204–3211 27270473 10.1210/jc.2016-1300

[47] Mehran L, Amouzegar A, Bakhtiyari M, Mansournia MA, Rahimabad PR, Tohidi M, Azizi F 2017 Variations in serum free thyroxine concentration within the reference range predicts the incidence of metabolic syndrome in non-obese adults: a cohort study. Thyroid 27:886–893 28486021 10.1089/thy.2016.0557

[48] Roos A, Bakker SJ, Links TP, Gans RO, Wolffenbuttel BH 2007 Thyroid function is associated with components of the metabolic syndrome in euthyroid subjects. J Clin Endocrinol Metab 92:491–496 17090642 10.1210/jc.2006-1718

[49] Garduño-Garcia J, Alvirde-Garcia U, López-Carrasco G, Mendoza M, Mehta R, Arellano-Campos O, Choza R, Sauque L, Garay-Sevilla ME, Malacara JM, Gomez-Perez FJ, Aguilar-Salinas CA 2010 TSH and free thyroxine concentrations are associated with differing metabolic markers in euthyroid subjects. Eur J Endocrinol 163:73–278 10.1530/EJE-10-0312 20516204

[50] Lin SY, Wang YY, Liu PH, Lai WA, Sheu WH 2005 Lower serum free thyroxine levels are associated with metabolic syndrome in a Chinese population. Metabolism 54:1524–1528 16253643 10.1016/j.metabol.2005.05.020

[51] Waring AC, Rodondi N, Harrison S, Kanava AM, Simonsick EM, Milkovic I, Satterfield S, Newman AB, Bauer DC; for the Health, Aging and Body Composition (Health ABC) Study 2012 Thyroid function and prevalent and incident metabolic syndrome in older adults: the health, aging, and body composition study. Clin Endocrinol (Oxf) 76:911–918 22187968 10.1111/j.1365-2265.2011.04328.x PMC3334430

[52] Shon HS, Jung ED, Kim SH, Lee JH 2008 Free T4 is negatively correlated with body mass index in euthyroid women. Korean J Intern Med 23:53–57 18646506 10.3904/kjim.2008.23.2.53 PMC2686972

[53] Makepeace AE, Bremmer AP, O'Leary P, Leedman PJ, Feddema P, Michelangeli V, Walsh JP 2008 Significant inverse relationship between serum free T4 concentration and body mass index in euthyroid subjects: differences between smokers and non-smokers. Clin Endocrinol (Oxf) 69:648–652 18346211 10.1111/j.1365-2265.2008.03239.x

[54] Chaker L, Ligthart S, Korevaar TI, Hofman A, Franco OH, Peeters RP, Deghan A 2016 Thyroid function and risk of type 2 diabetes: a population cohort study. BMC Med 14:150 27686165 10.1186/s12916-016-0693-4 PMC5043536

[55] Jun JE, Jee JH, Bae JC, Jin S-M, Hur KY, Lee M-K, Kim TH, Kim SW, Kim JH 2017 Association between changes in thyroid hormones and incident type 2 diabetes: a seven-year longitudinal study. Thyroid 27:29–38 27809684 10.1089/thy.2016.0171

[56] Oh H-S, Kwon H, Ahn J, Song E, Park S, Kim M, Han M, Jeon MJ, Kim WG, Kim WB, Shong YK, Rhee E-J, Kim TY 2018 Association between thyroid dysfunction and lipid profiles differs according to age and sex: results from the Korean National Health and Nutrition Survey. Thyroid 28:849–856 29779453 10.1089/thy.2017.0656

[57] Kim HH, Bae JC, Park HK, Byun DW, Suh K, Yoo MH, Kim JH, Min Y-K, Kim SW, Chung JH 2016 Triiodothyronine levels are independently associated with metabolic syndrome in euthyroid middle-aged subjects. Endocrinol Metab (Seoul) 31:311–319 27184017 10.3803/EnM.2016.31.2.311 PMC4923416

[58] Strollo F, Carucci I, More M, Marico G, Strollo G, Masini MA, Gentile S 2012 Free triiodothyronine and cholesterol levels in euthyroid elderly T2DM patients. Int J Endocrinol 2012:1–7 10.1155/2012/420370 PMC3438739 22973308

[59] Svare A, Nilsen TI, Bjøro T, Asvold BO, Langhammer A 2011 Serum TSH related to measures of body mass: longitudinal data from the HUNT Study, Norway. Clin Endocrinol (Oxf) 74:769–775 21521278 10.1111/j.1365-2265.2011.04009.x

[60] Proces S, Delgrange E, Vander Borght T, Donckier J, Donckier JE 2001 Minor alterations in thyroid-function tests associated with diabetes and obesity in outpatients without known thyroid illness. Acta Clinica Belgica 56:86–90 11383317 10.1179/acb.2001.015

[61] Wolide AD, Zawdie B, Alemayehu T, Tadesse S 2017 Association between thyroid hormone parameters and dyslipidemia among type 2 diabetes mellitus parameters: comparative cross-sectional study. Diab Metab Syndr Suppl 1:S257–S262 10.1016/j.dsx.2016.12.041 28057507

[62] Temizkan S, Balafoulou B, Ozderya A, Avci M, Aydin K, Karaman S, Sargin M 2016 Effects of thyrotrophin, thyroid hormones and thyroid antibodies on metabolic parameters in a euthyroid population with obesity. Clin Endocrinol 85:616–623 10.1111/cen.13095 27150556

[63] Jain RB 2017 Associations between the levels of thyroid hormones and lipid/lipoprotein levels: data from national Health and Nutrition Examination Survey 2007–2012. Environ Toxicol Pharmacol 53:133–144 28549315 10.1016/j.etap.2017.05.002

[64] Boekholdt SM, Titan SM, Wiersinga WM, Chatterjee K, Basart DCG, Luben R, Wareham NJ, Khaw K-T 2009 Initial thyroid status and cardiovascular risk factors: the Epic-Norfolk prospective population study. Clin Endocrinol 72:404–410 10.1111/j.1365-2265.2009.03640.x 19486022

[65] Udenze I, Nnaji I, Oshodi T 2014 Thyroid function in adults with metabolic syndrome. Pan Afr Med J 18:352 25574328 10.11604/pamj.2014.18.352.4551 PMC4282811

[66] Elgazar EH, Esheba NE, Shalaby SA, Mohamed WF 2019 Thyroid dysfunction prevalence and relation to glycemic control in patients with type 2 diabetes mellitus. Diabetes Metab Syndr 13:2513–2517 31405670 10.1016/j.dsx.2019.07.020

[67] Vadiveloo T, Donnan PT, Cochrane L, Leese G 2011 The Thyroid Epidemiology, Audit, and Research Study (TEARS): morbidity in patients with endogenous subclinical hyperthyroidism. J Clin Endocrinol Metab 96:1344–1351 21346066 10.1210/jc.2010-2693

[68] Choi HJ, Byun MS, Yi D, Sohn BK, Lee JH, Kim YK, Lee DY; KBASE Research Group 2017 Associations of thyroid hormone levels with in vivo Alzheimer's disease pathologies. Alzheimers Res Ther 9:64 28818092 10.1186/s13195-017-0291-5 PMC5561599

[69] Volpato S, Guralnik JM, Fried LP, Remalay AT, Cappola AR, Launer LJ 2002 Serum thyroxine level and cognitive decline in older women. Neurology 58:1055–1061 11940692 10.1212/wnl.58.7.1055

[70] Choi HY, Choe YM, Byun MS, Sohn BK, Baek H, Yi D, Han JY, Woo JI, Lee DY 2015 Associations between serum thyroid hormone and cerebral amyloidosis in cognitively diverse elderly. Alzheimers Dementia 11:S648–S649

[71] de Jong FD, Heijer T, Visser TJ, de Rijke YB, Drexhage HA, Hoffman A, Breteler MMB 2006 Thyroid hormones, dementia, and atrophy of the medial temporal lobe. J Clin Endocrinol Metab 91:2569–2573 16636121 10.1210/jc.2006-0449

[72] Tan ZS, Beiser A, Ramachandran RS, Au R, Auerbach S, Kiel DP, Wolf PA, Seshadri S 2009 Thyroid function and the risk of Alzheimer's disease: the Framingham Study. Arch Int Med 168:1514–1520 10.1001/archinte.168.14.1514 PMC2694610 18663163

[73] Yeap BB, Alfonso H, Chubb SA, Puri G, Hankey GJ, Flicker L, Almeida OP 2012 Higher free thyroxine levels predict increased incidence of dementia in older men: the Health in Men Study. J Clin Endocrinol Metab 97:E2230–E2237 22977271 10.1210/jc.2012-2108

[74] Chaker L, Wolters FJ, Korevaar TI, Hofman A, van der Lugt A, Koudstaal PJ, Franco OH, Deghan A, Vernooij MW, Peeters RP, Ikram MA 2016 Thyroid function and the risk of dementia: the Rotterdam study. Neurology 87:1688–1695 27638924 10.1212/WNL.0000000000003227

[75] Ittermann T, Wittfeld K, Nauck M, Bülow R, Hosten N, Völzke H, Grabe HJ 2018 High thyrotropin is associated with reduced hippocampal volume in a population-based study from Germany. Thyroid 28:1434–1442 30259797 10.1089/thy.2017.0561

[76] Yeap BB, Alfonso H, Chubb SAP, Walsh JP, Hankey GJ, Almeida OP, Flicker L 2012 Higher free thyroxine levels are associated with frailty in older men: the Health In Men Study. Clin Endocrinol 76:741–748 10.1111/j.1365-2265.2011.04290.x 22077961

[77] Bano A, Chaker L, Schoufour J, Ikram MA, Kavousi M, Franco OH, Peeters RP, Mattace-Raso FUS 2018 High circulating free thyroxine levels may increase the risk of frailty: the Rotterdam study. J Clin Endocrinol Metab 103:328–335 29126162 10.1210/jc.2017-01854

[78] van den Beld AW, Visser TJ, Feelders RA, Grobbee DE, Lamberts SWJ 2005 Thyroid hormone concentrations, disease, physical function, and mortality in elderly men. J Clin Endocrinol Metab 90:6403–6409 16174720 10.1210/jc.2005-0872

[79] Gussekloo J, van Exel E, de Graen AJM, Meinders AE, Frölich M, Westendorp RGJ 2004 Thyroid status, disability and cognitive function, and survival in old age. JAMA 292:2591–2599 15572717 10.1001/jama.292.21.2591

[80] Yeap BB, Alfonso H, Hankey GJ, Flicker L, Golledge J, Norman PE, Chubb SAP 2013 Higher free thyroxine levels are associated with all-cause mortality in euthyroid older men: the Health In Men Study. Eur J Endocrinol 169:401–408 23853210 10.1530/EJE-13-0306

[81] Van de Ven AC, Netea-Maier RT, de Vegt F, Ross HA, Sweep HA, Sween FC, Kiemeney LA, Smit JW, Hermus AR, den Heijer M 2014 Associations between thyroid function and mortality: the influence of age. Eur J Endocrinol 171:183–191 24801590 10.1530/EJE-13-1070

[82] Inoue K, Tsujimoto T, Saito J, Sugiyama T 2016 Association between serum thyrotropin levels and mortality among euthyroid adults in the United States. Thyroid 26:1457–1465 27539006 10.1089/thy.2016.0156

[83] Selmer C, Olesen JB, Hansen ML, von Kappelgaard LM, Madsen JC, Hansen PR, Pedersen OD, Faber J, Torp-Pederson C, Gislason GH 2014 Subclinical and overt thyroid dysfunction and the risk of all-cause mortality and cardiovascular events: a large population study. J Clin Endocrinol Metab 99:2372–2382 24654753 10.1210/jc.2013-4184

[84] Moon S, Kim MJ, Yu JM, Yoo HJ, Park YJ 2018 Subclinical hypothyroidism and the risk of cardiovascular disease and all-cause mortality: a meta-analysis of prospective cohort studies. Thyroid 28:1101–1110 29978767 10.1089/thy.2017.0414

[85] Razvi S, Weaver JU, Vanderpump MP, Pearce SH 2010 The incidence of ischemic heart disease and mortality in people with subclinical hypothyroidism: reanalysis of the Whickham Survey cohort. J Clin Endocrinol Metab 95:1734–1740 20150579 10.1210/jc.2009-1749

[86] Chaker L, van den Berg ME, Niemeijer MN, Franco OH, Deghan A, Hofman A, Rijnbeek PR, Deckers JW, Eijgelsheim M, Stricker BHC, Peeters RP 2016 Thyroid function and sudden cardiac death: a prospective study. Circulation 134:713–722 27601558 10.1161/CIRCULATIONAHA.115.020789

[87] Asvold BO, Bjøro T, Nilsen TI, Gunnell D, Vatten LJ 2008 Thyrotropin levels and risk of fatal coronary heart disease: the HUNT study. Arch Int Med 168:855–860 18443261 10.1001/archinte.168.8.855

[88] Walsh JP, Bremner AP, Bulsara MK, O'Leary P, Leedman PJ, Feddema P, Michelangeli V 2005 Subclinical thyroid dysfunction as a risk factor for cardiovascular disease. Arch Int Med 165:2467–2472 16314542 10.1001/archinte.165.21.2467

[89] Roef GL, Taes YE, Kaufman J-M, Van Daele CM, De Buyzere ML, Gillebert TC, Rietzschel ER 2013 Thyroid hormone levels within reference range are associated with heart rate, cardiac structure, and function in middle aged men and women. Thyroid 23:947–954 23339744 10.1089/thy.2012.0471 PMC3752520

[90] Rodondi N, Bauer DC, Cappola AR, Cornuz J, Robbins J, Fried LP, Ladenson PW, Vittinghoff E, Gottdiener JS, Newman AB 2008 Subclinical thyroid dysfunction, cardiac function and the risk of heart failure: the Cardiovascular Health Study. J Am Coll Cardiol 52:1152–1159 18804743 10.1016/j.jacc.2008.07.009 PMC2874755

[91] Peixoto de Miranda EJF, Bittencourt MS, Staniak HL, Sharovsky R, Pereira AC, Foppa M, Santos IS, Lotufo PA, Benseñor IM 2018 Thyrotropin and free thyroxine levels and coronary artery disease: cross-sectional analysis of the Brazilian longitudinal Study of Adult Health (ELSA-Brasil). Braz J med Biol Res 51:e7196 29561960 10.1590/1414-431X20177196 PMC5875905

[92] Vrijkotte TGM, Hrudey E, Twickler MB 2017 Early maternal thyroid function during gestation is associated with fetal growth, particularly in male newborns. J Clin Endo Metab 102:1059–1066 10.1210/jc.2016-3452 28359096

[93] Korevaar TIM, Schalekamp-Timmermans S, de Rijke YB, Visser WE, Visser W, de Muinck Keizer-Schrama SMPF, Hofman A, Ross HA, Hooijkaas H, Tiemeier H, Bongers-Schokking JJ, Jaddoe VW, Visser TJ, Steegers EA, Medici M, Peeters RP 2013 Hypothyroxinemia and TPO-antibody positivity are risk factors for premature delivery: the generation R study. J Clin Endocrinol Metab 98:4382–4390 24037884 10.1210/jc.2013-2855

[94] Medici M, de Rijke YB, Peeters RP, Visser W, de Muink Keizer-Schrama SM, Jaddoe VV, Hofman A, Hooijkaas H, Steegers EA, Tiemeiei H, Bongers-Schokking JJ, Visser TJ 2012 Maternal early pregnancy and newborn thyroid hormone parameters: the Generation R study. J Clin Endocrinol Metab 97:646–652 22162477 10.1210/jc.2011-2398

[95] Cleary-Goldman J, Malone FD, Lambert-Messerlain G, Sullivan L, Canick J, Porter TF, Luthy D, Gross S, Bianchi DW, D'Alton ME 2008 Maternal thyroid hypofunction and pregnancy outcome. Obstet Gynecol 112:85–92 18591312 10.1097/AOG.0b013e3181788dd7 PMC4949950

[96] Breathnach FM, Donnelly J, Cooley SM, Geary M, Malone FD 2013 Subclinical hypothyroidism as a risk factor for placental abruption: evidence from a low-risk primigravid population. Aust N Z J Obstetr Gynaecol 53:553–560 10.1111/ajo.12131 24111733

[97] Ashoor G, Maiz N, Rotas M, Jawdat F, Nicolaides KH 2010 Maternal thyroid function at 11–13 weeks of gestation and subsequent fetal death. Thyroid 20:989–993 20718684 10.1089/thy.2010.0058

[98] Knight BA, Shields BM, Hattersley AT, Vaidya B 2016 Maternal hypothyroxinaemia in pregnancy is associated with obesity and adverse maternal metabolic parameters. Eur J Endocrinol 174:51–57 26586839 10.1530/EJE-15-0866 PMC4761956

[99] Li Y, Shan Z, Teng W, Yu X LI Y, Fan C, Teng X, Guo R, Wang H, Li J, Chen Y, Wang W, Chawinga M, Zhang L, Yang L, Zhao Y, Hua T 2010 Abnormalities of maternal thyroid function during pregnancy affect neuropsychological development of their children at 25–30 months. Clin Endocrinol 72:825–829 10.1111/j.1365-2265.2009.03743.x 19878506

[100] Jackson K, Cooper DS 2019 Subclinical hypothyroidism and thyroid autoimmunity are associated with preterm delivery in an individual participant meta-analysis. Clin Thyroidol 31:410–416

[101] Rotondi M, Leporati P, La Manna A, Pirali B, Mondello T, Fonte R, Magri F, Chiovato L 2009 Raised serum TSH levels in patients with morbid obesity: is it enough to diagnose subclinical hypothyroidism? Eur J Endocrinol 160:403–408 19073832 10.1530/EJE-08-0734

[102] Rotondi M, Magri F, Chiovato L 2011 Thyroid and obesity: not a one-way interaction. J Clin Endocrinol Metab 96:344–346 21296993 10.1210/jc.2010-2515

[103] Matzen LE, Kvetny J, Pedersen KK 1989 TSH, thyroid hormones and nuclear binding of T3 in mononuclear blood cells from obese and non-obese women. Scand J Clin Lab Invest 49:249–253 2500700

[104] Köhrle J 1990 Thyrotropin (TSH) action on thyroid hormone deiodination and secretion: one aspect of thyrotropin regulation of thyroid cell biology. Horm Metab Res Suppl 23:18–28 2210628

[105] Ross DS 2019 Thyroid function in nonthyroidal illness. Cooper DS (ed) Waltham, MA: UpToDate, Inc. Available at https://www.uptodate.com (accessed 917, 2018)

[106] Fliers E, Kalsbeek A, Boelen A 2014 Beyond the fixed setpoint of the hypothalamus-pituitary-thyroid axis. Eur J Endocrinol 171:R197–R208 25005935 10.1530/EJE-14-0285

[107] Ellervik C, Roselli C, Christophersen IE, Alonso A, Pietzner M, Sitlani CMO, Trompet S, Arking DE, Geelhoed B, Guo X, Kleber ME, Lin HJ, Lin H, MacFarlane P, Selvin E, Shaffer C, Smith AV, Verweij N, Weiss S, Cappola AR, Dörr M, Gudnason V, Heckbert S, Mooijaart S, März W, Psaty BM, Ridker PM, Roden D, Stott DJ, Völzke H, Benjamin EJ, Delgado G, Ellinor P, Homuth G, Köttgen A, Jukema JW, Lubitz SA, Mora S, Rienstra M, Rotter JI, Shoemaker MB, Sotoodehnia N, Taylor KD, van der Harst P, Albert CM, Chasman DI 2019 Assessment of the relationship between genetic determinants of thyroid function and atrial fibrillation: a Mendelian randomisation study. JAMA Cardiol 4:144–152 30673084 10.1001/jamacardio.2018.4635 PMC6396813

[108] Larsson SC, Allara E, Mason AM, Michaëlsson K, Burgess S 2019 Thyroid function and dysfunction in relation to 16 cardiovascular diseases: a Mendelian randomisation study. Circ Genom Precis Med 12:e002468 30702347 10.1161/CIRCGEN.118.002468 PMC6443057

[109] Taylor PN, Richmond R, Davies N, Sayers A, Stevenson K, Woltersdorf W, Taylor A, Groom A, Northstone K, Ring S, Okosieme O, Rees A, Nitsch D, Williams GR, Smith GD, Gregory JW, Timpson NJ, Tobias JH, Dayan CM 2016 Paradoxical relationship between body mass index and thyroid hormone levels: a study using Mendelian randomization. J Clin Endocrinol Metab 101:730–738 26595101 10.1210/jc.2015-3505 PMC4880123

[110] DeGroot LJ 2000 Graves' disease and the manifestations of thyrotoxicosis. In: Feingold KR, Anawalt B, Boyce A, Chrousos G, Dungan K, Grossman A, Hershman JM, Kaltsas G, Koch C, Kopp P, Korbonits M, McLachlan R, Morley JE, New M, Perreault L, Purnell J, Rebar R, Singer F, Trence DL, Vinik A, Wilson DP (eds) Endotext [Internet]. South Dartmouth, MA: MDText.c0m, Inc. Available at https://www.ncbi.n1m.nih.gov/books/NBK285567 (accessed 711, 2015)

[111] Stabouli S, Papakatsika S Kotsis V 2010 Hypothyroidism and hypertension. Expert Rev Cardiovasc Ther 8:1559–1565 21090931 10.1586/erc.10.141

[112] Ryödi E, Salmi J, Jaatnen P, Huhtala H, Saaristo R, Välimäki M, Auvinen A, Metso S 2013 Cardiovascular morbidity and mortality in surgically treated hyperthyroidism—a nation-wide cohort study with a long-term follow-up. Clin Endocrinol 80:743–750 10.1111/cen.12359 24304446

[113] Gorka J, Taylor-Gjevre RM, Arnason T 2013 Metabolic and clinical consequences of hyperthyroidism on bone density. Int J Endocrinol 2013:1–11 10.1155/2013/638727 PMC3736466 23970897

[114] Fukui T, Hasegawa Y, Takenaka H 2001 Hyperthyroid dementia: clinicoradiological findings and response to treatment. J Neurol Sci 184:81–88 11231037 10.1016/s0022-510x(00)00487-1

[115] Duntas LH 2002 Thyroid disease and lipids. Thyroid 12:287–293 12034052 10.1089/10507250252949405

[116] Nikkilä EA, Kekki M 1972 Plasma triglyceride metabolism in thyroid disease. J Clin Invest 51:2103–2114 4341014 10.1172/JCI107017 PMC292367

[117] Prisant LM, Gujral JS, Mulloy AL 2007 Hyperthyroidism: a secondary cause of isolated hypertension. J Cin Hypertens 8:2103–2114 10.1111/j.1524-6175.2006.05180.x PMC8109671 16896276

[118] Anantarapu S, Vaikkahura S, Sachan A, Phaneendra BV, Suchitra MM, Reddy AP, Epuri S, Mukka A, Vemvakam D 2015 Effects of thyroid hormone replacement on glycated hemoglobin levels in non-diabetic subjects with overt hypothyroidism. Arch Endocrinol Metab 59:495–500 26421666 10.1590/2359-3997000000065

[119] Andersen SL, Olsen J, Wu CS, Laurberg P 2014 Spontaneous abortion, stillbirth and hyperthyroidism: a Danish population-based study. Eur Thyroid J 3:164–172 25538898 10.1159/000365101 PMC4224233

[120] Sahay RK, Sri Nagesh V 2012 Hypothyroidism in pregnancy. Indian J Endocrinol Metab 16:364–370 22629500 10.4103/2230-8210.95667 PMC3354841

[121] Calvo RM, Jauniaux E, Gulbis B, Asunción M, Gervy C, Contempré B, Morreale de Escobar G 2002 Fetal tissues are exposed to biologically relevant free thyroxine concentrations during early phases of development. J Clin Endocrinol 87:1768–1777 10.1210/jcem.87.4.8434 11932315

[122] Moeller LC, Führer D 2013 Thyroid hormone, thyroid hormone receptors, and cancer: a clinical perspective. Endocr Relat Cancer 20:R19–R29 23319493 10.1530/ERC-12-0219

[123] Theodossiou C, Schwarzenberger P 2000 Propylthiouracil reduces xenograft tumour growth in athymic nude mouse prostate cancer model. Am J Med Sci 319:96–99 10698093 10.1097/00000441-200002000-00005

[124] Fernandez-Ruocco J, Gallego M, Rodriguez-de-Yurre A, Zayas-Arrabal J, Echeazarra L, Alquiza A, Fernández-López V, Rodriguez-Robledo JM, Britto O, Schleier Y, Sepulveda M, Oshiyama NF, Vila-Petroff M, Bassani RA, Medel EH, Casis O 2019 High thyrotropin is critical for cardiac electrical remodelling and arrhythmia vulnerability in hypothyroidism. Thyroid 29:934–945 31084419 10.1089/thy.2018.0709 PMC6648210

[125] Fitzgerald SP, Bean NG, Fitzgerald LN 2017 Population data indicate that thyroid regulation is consistent with an equilibrium-point model, but not with a set point model. Temperature (Austin) 4:114–116 28680925 10.1080/23328940.2017.1281370 PMC5489013

[126] Walsh JP 2011 Setpoints and susceptibility: do small differences in thyroid function really matter? Clin Endocrinol 75:158–159 10.1111/j.1365-2265.2011.04036.x 21521305

[127] Fitzgerald SP, Bean NG 2018 Thyroid stimulating hormone (TSH) autoregulation reduces variation in the TSH response to thyroid hormones. Temperature (Austin) 5:380–389 30574530 10.1080/23328940.2018.1513110 PMC6298488

[128] De Leo S, Lee SY, Braverman LE 2016 Hyperthyroidism. Lancet 388:906–918 27038492 10.1016/S0140-6736(16)00278-6 PMC5014602

[129] Lacroix A, Feelders RA, Stratakis CA, Nieman LK 2015 Cushing's syndrome. Lancet 386:913–927 26004339 10.1016/S0140-6736(14)61375-1

[130] Sheehan MT 2016 Biochemical testing of the thyroid: TSH is the best and, oftentimes, only test needed—a review for primary care. Clin Med Res 14:83–92 27231117 10.3121/cmr.2016.1309 PMC5321289

